# The patients’ experience of a bladder cancer diagnosis: a systematic review of the qualitative evidence

**DOI:** 10.1007/s11764-017-0603-6

**Published:** 2017-02-17

**Authors:** Amanda J. Edmondson, Jacqueline C. Birtwistle, James W.F. Catto, Maureen Twiddy

**Affiliations:** 1 Centre for Applied Research in Health, University of Huddersfield, Huddersfield, UK; 2 Institute of Health Sciences, University of Leeds, Leeds, UK; 3 Academic Urology Unit, University of Sheffield, Sheffield, UK

**Keywords:** Bladder cancer, Diagnosis, Treatment, Cystectomy, Quality of life, Patients’ experience

## Abstract

**Purpose:**

Bladder cancer (BC) is a common disease with disparate treatment options and variable outcomes. Despite the disease’s high prevalence, little is known of the lived experience of affected patients. National patient experience surveys suggest that those with BC have poorer experiences than those with other common cancers. The aim of this review is to identify first-hand accounts of the lived experiences of diagnosis through to survivorship.

**Method:**

This is a systematic review of the qualitative evidence reporting first-hand accounts of the experiences of being diagnosed with, treated for and surviving bladder cancer. A thematic analysis and ‘best-fit’ framework synthesis was undertaken to classify these experiences.

**Results:**

The inconsistent nature of symptoms contributes to delays in diagnosis. Post-diagnosis, many patients are not actively engaged in the treatment decision-making process and rely on their doctor’s expertise. This can result in patients not adequately exploring the consequences of these decisions. Learning how to cope with a ‘post-surgery body’, changing sexuality and incontinence are distressing. Much less is known about the quality of life of patients receiving conservative treatments such as Bacillus Calmette-Guerin (BCG).

**Conclusions:**

The review contributes to a greater understanding of the lived experience of bladder cancer. Findings reflect a paucity of relevant literature and a need to develop more sensitive patient-reported outcome measures (PROMs) and incorporate patient-reported outcomes in BC care pathways.

**Implications for cancer survivors:**

Collective knowledge of the patients’ self-reported experience of the cancer care pathway will facilitate understanding of the outcomes following treatment.

**Electronic supplementary material:**

The online version of this article (doi:10.1007/s11764-017-0603-6) contains supplementary material, which is available to authorized users.

## Introduction

Bladder cancer (BC) is the seventh most common cancer in the world [1] and is one of the most expensive to manage [2]. The disease is more common in males than females, reflecting the main etiological risk factors, i.e. cigarette smoking and occupational carcinogen exposure [3]. Despite advances in the epidemiology and treatment, relatively little is known about the experience of patients diagnosed with BC [4, 5]. Patient surveys have shown that the experience of those with BC is one of the poorest when compared to other cancers. Potential explanations for this include absence of care planning, emotional support and poor post-discharge care [6]. These factors may be compounded by the male predominance of BC and the tendency of men to internalise their illness behaviour [7].

Whilst most cancers affect the well-being and quality of life (QoL) of diagnosed individuals and their caregivers, the QoL for BC patients is not well known due to a lack of disease and treatment-specific validated measure(s) and a lack of large-scale analyses [8–13]. Where data are available, reports are often restricted to small samples post-treatment [14, 15] and so limit the understanding of the BC patients’ experience following diagnosis and pre-treatment experience of care (reviewed in [4]). Developing new measures which identify care needs across the patients’ pathway will help improve clinical practice and assist them in the early stages of their diagnosis and treatment decision making [16]. Given that recent reviews have focussed upon quantitative data (e.g. [4]), we undertook a systematic review of the current status of qualitative data in patients with BC.

In 2010, the National Cancer Survivor Initiative (NCSI) published a ‘vision’ document [5] that reported a number of key shifts required in the approach to care for people living with and beyond cancer. One key vision was moving the focus from measuring clinical activity to measuring experience, concerns and outcomes for cancer survivors through routine use of patient-reported outcome measures (PROMs). The value of qualitative research in the development of PRO measures has been recognised for some time. For example, Duncan et al. [17] recently conducted a synthesis of the qualitative evidence to examine the QoL domains from the patients’ perspective to facilitate PROM development in five specific health conditions. This article also presents a systematic search of the qualitative literature and a ‘best-fit’ framework synthesis [18] to classify and enhance the understanding of the experiences of BC from the patients’ perspective. Findings may also assist future PROM development.

## Method

**Fig. 1:**
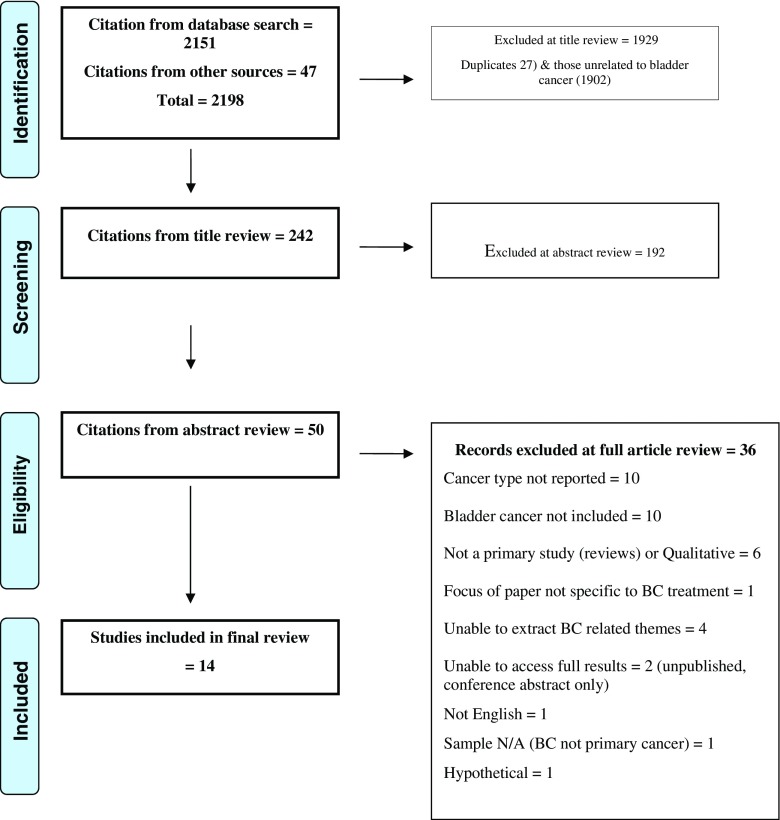
Flow chart of included and excluded studies

Preferred Reporting Items for Systematic Reviews and Meta-Analyses (PRISMA) guidelines were followed, including reference to the 27-item checklist (where applicable) and four-phase item flow diagram [19] (see Fig. 1).

### Literature search

Medline, PsycInfo, Embase, CINAHL, Cochrane Library, Global Health, Scopus, Pro Quest (Health & Medicine) and Google Scholar were searched for articles published between January 2000 and January 2016. A combination of keywords to describe the patient, patients’ experience, BC, treatment and research method was used (for example, to describe the patient [patient, cancer patient, surgical patient, hospitalised patient], their experience [acceptance, attitude, beliefs, opinion, satisfaction, QoL, quality of care, understanding, feelings], the cancer [BC, urinary bladder neoplasms, bladder, carcinoma], the treatment [intravesical immunotherapy, BCG vaccine, cystectomy, surgery] and the research method [interview, case studies, observations, focus group, thematic analysis], see online resource 1 for sample search strategy). In addition, reference lists and citations of included studies were scanned and specific urology journals were hand searched (Current Opinion in Urology, European Urology, Urology Practice, Journal of Urology, Urology, Scandinavian Journal of Urology and Nephrology and Scandinavian Journal of Urology and Asian Journal of Urology). Lead authors (identified from the searches/included studies) and conference abstracts (APOS, BPOS) were searched. English language restriction was applied to the search.

### Study selection

All primary studies detailing self-reported accounts of the experience of being diagnosed and treated for BC (primary cancer) were included in the review, meaning that results are based upon the narratives of individuals with BC. Studies that reported survey data only or hypothetical data were excluded. Two authors (AE and JB) reviewed the titles and abstracts to apply the inclusion criteria, and potentially eligible full-text articles were evaluated by AE, JB and MT for eligibility and quality. Each article was assessed for quality using the Critical Appraisal Skills Programme (CASP) assessment tool for qualitative research [20]. All studies were included in the review regardless of their quality rating, but the rating was used as an indication of the strength of the evidence and to inform standards required for future research into the qualitative experiences of people diagnosed with BC. Disagreements were resolved by discussion.

### Data synthesis

Data regarding population, age range, participant numbers, diagnosis, treatment, data collection approach and derived themes were extracted using a standardised form. A thematic analysis of all the identified lived experiences of BC, using a ‘best-fit framework synthesis’ [18], was undertaken starting with very similar themes to those described by Beitz and Zuzelo [21], which were experience of diagnosis, acute care and treatment, post-treatment and the new normal. These informed the framework as they encompassed the patients’ pathway from diagnosis to survivorship and adapting (or not) to life and thereby fit with the aims of this review. Initial allocation of experiences to the framework was undertaken by AE aided by discussion with JB and MT in uncertain cases.

## Results

### Manuscript selection

In total, 2198 manuscripts were identified, from which 14 eligible studies were selected (Fig. 1 and online resource 2). Most studies were North American (*n* = 9) or British (*n* = 3), with 1 from Italy and Sweden. Accounts of 270 participants, of which 188 (70%) were male and 76 (30%) were female (gender missing in one study, *n* = 6), with an age range from 33 to 86 years are reported. Diagnoses included non-muscle-invasive, muscle-invasive and metastatic BC. Treatments included radical cystectomy with various urinary diversions, systemic chemotherapy, radical radiation, transurethral resection and intravesical Bacillus Calmette-Guerin (BCG). At least half of the studies focus on the experiences of radical surgery patients, other papers did not report treatments provided, and only two papers focussed exclusively on BCG patients. Articles were scored for quality, with a mean quality score of 7 (range 5–9/9 points). Lower scores reflected an absence of ethical standard statements and risk or presence of bias during the process.

### Patients’ experience of diagnosis

Patients’ experience of diagnosis theme captures patients’ reported experience of being diagnosed with BC, including presenting symptoms, diagnostic process, pre-treatment consultations and the treatment decision-making process.

#### Diagnosing BC

Typical symptoms of BC were visible haematuria and altered urination patterns (urgency, frequency and dysuria) [21, 22]. Patients described haematuria as deceptive due to its painless and inconsistent nature [21]. The lack of pain and understanding about BC led to a delay in seeking help [21, 23, 24]. When help was sought, some felt frustrated that their symptoms were trivialised and misdiagnosed. For example, women presenting with blood in their urine were often met with ‘are you sure it’s not just your menstrual cycle?’ [21], reflecting the typical delay in referral for female patients with BC [25].

A typical response to diagnosis was shock, upset [21, 23] and devastation [26], followed by a fear of treatment [26] and an intense desire for a speedy intervention [21]. Some described feeling ‘scared to death’ and thought of their diagnosis as a death sentence [21, 23, 26]: ‘you’re sitting there thinking I’m going to die’ [23]. Hilton and Henderson [24] described this experience as ‘unknowing’—everything that patients know about their health is suddenly called into question and they may now worry that their overall health will deteriorate [26].

#### Treatment decision making

Making treatment decisions was perceived as very challenging. Patients described difficulty understanding medical explanations, treatment options and potential side effects [27] and felt uncomfortable making such decisions [21]. Cancer information became important, and patients reported receiving insufficient information about self-care after treatment (surgery), finances and insurance, and subsequently, many sought information via the internet and/or support groups [27]. Worries about survival, pain, reduced sexual function and change in body image (after surgery) were often not addressed. In one recent study, only 6 out of 30 patients reported discussing likely changes in sexual function following surgery during the treatment consultation [27]. Some patients also reported receiving conflicting treatment recommendations and felt that there was a bias towards particular treatments depending on the healthcare professional that they spoke to [21].

Berry and colleagues [28] explored how patients perceived and engaged in treatment decision making. They found patients expended significant effort in identifying the best healthcare provider ‘one of the things I’ve always kept as a reference point is where are the centers of excellence for various treatments?’ [28], even if this meant travelling a significant distance for treatment. In contrast, when considering treatment choice, almost half of patients were passive in the decision-making process and accepted the clinicians’ treatment recommendation without question, but this was not constructed as problematic by patients ‘it’s like, no you [clinician] tell me what to do’ [28] and was most common in patients with early-stage disease. Other patients sought information from the internet, family, friends and others with knowledge/personal experience to inform discussions with their clinician. A small number reported having complete control over the decision ‘at the end I [patient] was the only one who would make the decision’ [28]. Treatment choice was largely influenced by survival statistics, but other factors, such as treatment preferences [29], age and level of recreational and work activity, played an important role [28]: ‘I based it on the fact I’m 59 so it’s not like I’m 20 and I have to live with this bag for a hundred years’; ‘I’ve never had a period in my life where I wasn’t exercising so an ostomy bag was really not an option for me’ [28].

Unsurprisingly, open communication was a critical and reoccurring theme throughout the patients’ pathway, but particularly so in diagnosis and treatment consultations [23, 28, 30]. Early impressions of interpersonal aspects of patient care are important to the patient, in particular whether they feel that they are being treated as someone who matters and is worthy of care and being recognised and responded to as a unique individual with a particular social context [30]—‘I say yes it’s like being on first name terms with some of them…oh they get to know you and you get to know them’ [30]. Patients believed that it was important to have ‘a conversation’ with the clinician, where the options are discussed to ensure that the clinician understands the impact of treatment options on the patient’s life. The speed and momentum of diagnosis and treatment can result in patients feeling ill prepared, in particular for the side effects of treatment [28]. They wanted treatment plans to be clear, provided in a timely fashion and consistent from professional to professional [23].

### Patients’ experience of acute care and treatment

Patients’ experience of acute care and treatment theme captures patients’ experiences of preparing for treatment and their acute care.

#### Preparing for surgery

The psychological preparation for surgery can start weeks before admission [24, 31]. One patient described it as worse than the diagnosis; for her, the thought of the impending surgical procedure (vaginal reconstruction) was devastating and terrifying, and she felt uncertain whether she would ever be the same again [24]. Hilton and Henderson [24] described this experience of an impending bodily change as ‘metamorphosis’. Physical pre-surgical procedures were also captured in detail. For example, patients recalled the onerous procedures of bowel preparation prior to surgery [21], and neobladder reconstruction patients recalled being measured and ‘tattooed’ for stoma placement prior to their surgery, even though they were not expecting a stoma. This was described as unsettling, and for some, the fear of the change to self following surgery felt worse than the diagnosis [21, 24].

#### Waking up after surgery

Waking after surgery is described as a feeling of ‘alienation from the body’ [32]. This encapsulates the shock and disgust some patients’ experience in response to their stoma and numerous abdominal drains [21]. Simple acts of kindness are important ‘what a nice woman that was [nurse] when I woke up after my first operation when I opened my eyes she was sitting at my bed holding my hand now what do you think of that…that’s a good one’ [30].

#### Post-operative care

Hands-on training on patient’ stoma appliances and catheters begins in the acute recovery phase. This was a positive experience, but many felt that it should have continued after discharge [27]. Although aftercare was generally good, for some, post-operative pain was not well managed, with pain management regimes leaving patients feeling ‘knocked out’ or ‘mentally in the left field’ [21] and disorientated to time and place [21].

#### Patients’ experience of non-radical surgical treatment

Two studies captured non-radical surgical treatment experiences. Patients commonly reported short-lived related symptoms [22, 26]. Patients receiving BCG treatment reported abdominal pain and painful, urgent and more frequent urination [22]. Some also reported passing blood clots and blood in the urine, flu-like symptoms, fatigue and soreness at the catheter site [22]. Clark [22] interviewed patients who had undergone TURBT, and those patients described painful and urgent urination, knife-like stinging and passing blood clots—‘it was just the initial shock when you put that thing in, and the first time you go to the potty and urinate that hurt. That hurt like hell’. However, symptoms were generally temporary.

### Patients’ experience post-treatment

Patients’ experience post-treatment theme mostly captures the period shortly after treatment, during which patients experience immense change, and details the ways in which people learn to adapt to new, often distressing experiences.

Post-surgery recuperation was long and something that patients felt that they needed support with [21]. It was described by one patient as ‘the point I became a cancer survivor’ [24]. Hilton and Henderson [24] referred to this experience as ‘an unfolding path’—recuperation was a time of immense change, encompassing new experiences, new learning and adaptation both physically and psychologically [21, 23, 24]. Weight loss following surgery was common, and patients felt exhausted and weak on their return home. Although they felt unhappy about lost vitality, there was a sense of acceptance that they needed to pace their activities and some employed coping strategies, such as starting walking routines to regain strength [21].

Support of family and friends was especially valuable at this time, though paradoxically, this was a time when some experienced disappointment and difficulty with close relationships; a few patients reported feeling disappointed by the lack of support and felt as though they were being treated differently [23].

Patients’ experience of homecare was variable, and it was a lottery in terms of how much aftercare they might receive [23]. Knowledge and expertise to deliver homecare to patients following a radical cystectomy varied—‘the homecare, nobody, not one person knew or had any experience with this. They had experience with bowels but not bladder’ [23].

New experiences were often unexpected and distressing. For example, patients reported not being told how they could deal with incontinence. Many reported initiating their own strategies such as wearing pads at night, changing underwear style, only wearing black trousers so leakage would not show and establishing bladder schedules, for example, setting alarms to go off through the night to ensure regular voiding [21, 23], in the absence of education from healthcare services [23]. Some neobladder patients had to learn to self-catheterise; this felt easier than learning to void their neobladder. The mechanics of voiding the neobladder are very different to those of their original bladder and more redolent of defecation in that they needed to ‘force it out’ and ‘strain’ [21]. Self-catheterising for some, however, felt disgusting and was avoided [21].

### The new normal

Having experienced a period of immense change, this theme captures the next phase, referred to as the ‘new normal’ [21]. Here, patients describe their QoL post-treatment, i.e. their experience of adapting (or not) to new toileting characteristics, new ways of being sexual and living with the lifelong threat of cancer.

Quality of life (QoL) following treatment for BC was mixed for both surgical and non-surgical patients. Patients reported both negative and positive aspects, but they were also something fluid and they fluctuated over time [15, 29]. For example, Cerruto and colleagues [15] explored the QoL of a cross section of patients (1 year post-surgery up to 30 years) who had an ileal conduit following a radical cystectomy. They presented patient profiles at 1, 3, 5, 7 and plus 7 years post-surgery. One year post-surgery, QoL was reported to be good/unchanged for some, but for others, it was worse, with poor sleep and being dependent on others to manage their ostomy notable areas of concern. By 3 years, most reported having poor QoL; main areas of difficulty were continued dependence on partners to manage their ostomy, concern about leakage and smell of urine and subsequent decline of social activities. Worsening of QoL over time was reported for surgical and non-surgical populations and attributed to a decreasing optimism about recovery [15, 29] and for surgical patients, the overwhelming feeling of not being the same [15]. Loss of friendships and the detrimental impact on social life were also reported by Persson and Hellstrom [32], but they noted that these occurred quite soon after surgery when patients were faced with who, how to tell and how people would react.

By 5 years post-treatment, QoL had improved and patients reported feeling in a better state of health compared to pre-surgery. Cerruto and colleagues [15] attributed this improvement to adaptation. Patients reported feeling less dependent on partners; problems such as urinary leakage remained, but these were managed; ‘I don’t have anxiety about my condition, there are some precautions that should be observed, I must be careful that there are no leaks but it happens rarely in my case and I can live almost normally’ [15]. By 7 years post-surgery, social relations had recovered and activities of daily living felt less restricted. This finding was also supported by Foley et al. [33] who explored the cancer experience of long-term survivors and found over time that survivors had acquired a greater appreciation for life.

However, some concerns persisted over time; for surgical patients, this included a lack of sexual activity and physical complications such as hernias, urinary tract infections and peristomal skin lesions, which affect ostomy management and risk leakage [15]. Long-term effects of cancer were described by survivors as ‘a constant’ in their lives and as a reminder of their cancer [21, 34].

#### Accepting incontinence

Incontinence following surgery was generally permanent, and learning not to be embarrassed about leakage was key to successful management [21]. New routines to respond to new toileting characteristics were commonplace [21, 27]. Some patients described difficulties and subsequent adaptations related to returning to work. For example, finding a clean place to self-catheterise away from home was described as difficult, particularly for men. Male public toilets were often perceived as dirty, and sitting on the toilet seat was unfamiliar and frustrating. For some men, this resulted in a reluctance to travel or where necessary holding large volumes of urine to avoid using public toilets. For some, a change in toilet characteristics also extended to their bowels; some experienced chronic diarrhoea and unpredictable flatulence [21, 32]. Patients described locating toilets ahead of time as a protective strategy, and planning their toilet use became a major priority [21, 27]: ‘If you go to some function probably the first thing you seek out is the toilet’; ‘Life is normal. It’s almost as if it didn’t happen except for the inconvenience of having to sit and plan where I go based on having to go to the bathroom’ [21].

Despite understanding the importance of hydration, many surgical participants reported not drinking enough. For some, this was because of the need to subsequently empty their bladder, which meant staying closer to a toilet, which increased isolation as patients remained at home; for others, it was about managing continence, with some patients avoiding beer as this often resulted in night-time leakage [21].

Ongoing fears included leakage of gas and odour and visibility of the stoma [32]. Patients often selected different clothes to minimise visibility and damage to the stoma (e.g. wearing loose dresses, supporting the stoma with suspenders [32]). Concerns about visibility also resulted in changes to social activities, for example, avoidance of swimming pools [32].

#### Changing sexuality

Changes in sexuality were reported by men and women [27] who had undergone non-surgical [26, 35] and radical surgical treatment [21, 23, 35]. Non-radically treated patients usually reported a short period of abstinence due to fear of contamination of their partner with the treating agents [26, 35], but for some, abstinence seemed more permanent ‘we don’t have sex because of that stuff they were putting in me. I still get an erection and masturbate and I don’t tell her about that but I do and when I come it doesn’t come out like it used to because of that irritation in there’ [35]. For radical surgical patients, despite having prior knowledge about the impact on sexual function, i.e. impotence for men and vaginal shortening/dryness for women, the reality was still a shock. Men in particular had been certain that it would not be the case for them and described impotence as a loss of their manhood, which led to other ways of achieving an erection [27, 35], but this was often met with disappointment [21]; ‘no more sex life, I feel destroyed physically, emotionally. Once I was a master of myself, now I depend on my wife. The surgery carried away all that I had’ [15]. For others, sexual relationships were re-established but in a different way; ‘BC has changed our sex life a bit, we still have sex but it’s different now, well obviously it’s different for me. Since I can’t have normal intercourse it’s a lot of foreplay but I enjoy that too. It’s not as good as it was before but it’s still pretty good, I bought a vibrator so she can still have orgasms—it changed the dynamic of sex, you know it’s more to make sure she has an orgasm’ [35].

Post-surgery, women reported physical and psychological concerns about sex [23, 27, 35], with the loss of physical intimacy commonly reported ‘sometimes it’s almost a platonic relationship’ [35]. The appearance of the stoma and the bag were of concern for some as they perceived it to be off-putting sexually [23, 27, 32, 35], with some fearing leakage from the stoma during sex [23]; ‘not in a million years would I let anybody close to me with this stoma and bag and all that, I’m disgusting. How it looks, I mean I have a bag of pee hanging in front of me, I find it revolting I’m sure anyone else would’ [23].

The degree of acceptance about loss of sexual function was reported to be influenced by age, stage of life and how much importance a couple placed on sex [23]. In contrast, re-establishing a sexual relationship after BC was influenced by good communication between partners [35]. Interestingly, despite how common sexual problems seem to be amongst BC survivors, very few sought professional assistance [27], with Mohamed and colleagues positing that this was perhaps due to the fact that many were grateful to be alive [27].

#### Living with the lifelong threat of cancer

‘Deal with it’ and ‘just take it as it comes’ attitudes were commonplace [21, 29, 33, 34]. Survivors reported being very aware that many people die from BC, and so, a stoical and optimistic attitude to new experiences soon developed [21, 23, 33]. Similarly, living each day and having a new found sense of appreciation for their life were also apparent and may be partly attributed to the perception of cancer as a lifelong threat [21, 27, 34]. Follow-up schedules proved to be a constant reminder of how fragile life can be [21, 34], and many survivors reported that support from family and friends had been vital throughout their journey with cancer [29, 34] (see online resource 3 for a preliminary conceptual framework of the patients’ experience of being diagnosed with BC through to survivorship).

## Discussion

This is the first systematic review of qualitative evidence focusing upon first-hand accounts of the lived experience of BC. The review identified the significant impact of this disease upon the patient and their next of kin and that currently, there is little attention paid to this by BC care practitioners. Most of the data reported events at the beginning (at diagnosis) and end (life after treatment) of the patients’ pathway, and there was an over-representation of patients undergoing radical surgery, when the majority of patients receive non-radical (conservative) treatments. As the concerns of those undergoing conservative treatments cannot be assumed to be the same as those of the RC population, the review highlights the need for more qualitative research to inform understanding of the experiences of this population.

Sexual concerns were especially common with an unmet need for information and support [21, 23, 26, 27, 32, 35], which support the findings of a recent PROM pilot report [36]. The fact that very few patients reported receiving help for sexual distress is a concern [27] and suggests perhaps that the shift towards exploring the patients’ holistic experience, in particular their sexual experience, as set out by the National Cancer Survivorship vision has not yet been reached [5]. The findings of the review suggest that health professionals need to be more proactive in eliciting areas of distress and, given the gender-specific concerns highlighted in this review, tailored interventions would be more appropriate [27].

Body image was an important concern for those undergoing surgery. Patients reported experiencing significant alteration to their body [32], and women in particular reported feeling unsexual [23, 27, 32, 35]. Visibility of the stoma was problematic and resulted in patients altering clothing and social activities [32]. Concerns about body image were also reported in the PROM pilot report, and the findings of this review evidence that conclusion [36]. Only one (excluded) study found body image not to be important [37], but this may be due to those authors asking patients to consider a future event (impact of surgery) on body image; it is not clear whether patients were specifically asked about appearance post-surgery, and given the duration of the interviews (16 min on average), it seems unlikely that any discussion would have been in-depth. Encouraging patients to reflect on their experiences in some depth enables a more valid disclosure of concerns [32], which might account for the disparate findings.

Less frequently described but nonetheless explored was patients’ experience of acute care and discharge, and this review highlights how some of the most basic acts of kindness, such as holding a patient’s hand on waking from surgery, can make such a difference to their experience.

This review identifies a relatively neglected area of cancer and the poor level of evidence in this field. It offers an understanding of the patients’ experience pre-diagnosis through to survivorship, complementing a recent BC PROM pilot [36] and ongoing work in BC QoL development to develop ways to comprehensively assess sexual [8] and body image issues in particular [38]. It also serves as a useful starting point for developing teaching/training materials. Knowledge of the patients’ experience from diagnosis through to survivorship and highlighting the challenges in reporting concerns are valuable to new and existing health professionals tasked with shifting the focus from clinical activity to patients’ experience. Finally, it is a response to patients’ hopes for professionals to better understand their experience and in particular the impact of bodily and sexual changes [23].

### Limitations

The review only included articles in the English language, and most of the included studies were carried out in North America (within a specific healthcare system). As such, it may limit the understanding of a more global picture of patients’ experience with BC. The review applied a date limitation from 2000 to present. Although this will have restricted our search and subsequently missed articles of relevance, it was an attempt to capture patients’ contemporary experiences of clinical services and treatments. In synthesising the data from all the studies, irrespective of their cancer stage and treatment, subtleties in relation to QoL, need, etc. associated with certain treatments or extent of illness will have been missed. Nevertheless, several themes identified in the review are expected to be common throughout the illness trajectory and helpful in addressing future care irrespective of stage/treatment.

### Recommendations

The findings of this review are relevant and important to the field but reflect a paucity of relevant literature. Prior to the development of any new measure, a clear conceptual framework is needed [39–41], and this review suggests that there are gaps in our understanding that need to be filled before high-quality, sensitive measure of QoL can be developed for this population. This article offers the beginnings of a conceptual framework (see online resource 3); however, to develop a robust framework, more research is needed. Future research should aim to improve reporting of qualitative findings relating to BC, should include larger numbers of patients (and caregivers) receiving non-radical treatments and should include longitudinal studies to capture change over time. With this in mind, authors are now undertaking longitudinal surveys into the QoL in patients being treated for and after a diagnosis of BC, e.g. the OTIS study [http://www.abdn.ac.uk/hsru/research/assessment/interventional/otis/].

### Implications for practice

The review highlights that a better understanding of the patients’ experience throughout each stage of their pathway could be gleaned. It is clear that patients’ experience varies and some require more or less support than others at different points along the their pathway. Support and informational needs may be gender specific and may differ in intensity, for example, for those who may not have support from family and/or friends.

## Conclusion

The findings contribute, through a qualitative synthesis, to a greater understanding of the lived experience of BC. The review has pooled the evidence making it more accessible to individual centres where numbers of patients with BC may be small, thus restricting knowledge of the full effects of cancer for this group of patients. This might also explain why the patients’ experience for this group of patients has received less attention, compared to other cancer types (e.g. breast). It is noteworthy that these experiences are identified from self-reports, which suggest that discussion of them might be incorporated into the clinical pathway when appropriate. The findings identify the impact of BC upon the lived experience and suggest a need to embed PROMs within care pathways and to encourage care providers to understand their importance.

## Electronic supplementary material

ESM 1 (DOCX 21.1 kb).

ESM 2 (DOCX 31.7 kb).

ESM 3 (DOCX 34.0 kb).
