# Supplementary material for: The patients’ experience of a bladder cancer diagnosis: a systematic review of the qualitative evidence
Source: J Cancer Surviv. 2017 Feb 17;11(4):453–61. doi: 10.1007/s11764-017-0603-6 (PMC5500680; doi:10.1007/s11764-017-0603-6)
Supplement: Supplementary file 1 — (DOCX 21.1 kb) [file 11764_2017_603_MOESM1_ESM.docx]

**Online Resource 1**

Example search Strategy: Psychinfo

exp Hospitalized Patients/ or exp Patients/ or exp Medical Patients/ or exp Surgical Patients/

2. exp Neoplasms/ or exp Treatment/ or exp Bladder/ or bladder cancer treatment.mp. or exp "Side Effects (Treatment)"/

3. exp Immunization/ or BCG vaccine.mp.

4. exp Neoplasms/ or exp Surgery/ or exp Bladder/ or cystectomy.mp.

5. exp Bladder/ or exp Neoplasms/ or bladder cancer.mp.

6. belief*.mp. or exp False Beliefs/

7. experience.mp.

8. opinion.mp. or exp Attitudes/

9. exp Satisfaction/ or satisfaction.mp.

10. quality of life.mp. or exp "Quality of Life"/

11. acceptance.mp.

12. health knowledge.mp. or exp Health Knowledge/

13. understanding.mp. or exp Comprehension/

14. exp "Quality of Life"/ or exp Illness Behavior/ or exp "Physical Illness (Attitudes Toward)"/ or exp Health Behavior/ or illness representation.mp. or exp Patients/

15. exp Methodology/ or exp Qualitative Research/ or qualitative.mp.

16. 2 or 3 or 4 or 5

17. 6 or 7 or 8 or 9 or 10 or 11 or 12 or 13 or 14

18. 1 and 15 and 16 and 17
