# Supplementary material for: The patients’ experience of a bladder cancer diagnosis: a systematic review of the qualitative evidence
Source: J Cancer Surviv. 2017 Feb 17;11(4):453–61. doi: 10.1007/s11764-017-0603-6 (PMC5500680; doi:10.1007/s11764-017-0603-6)
Supplement: Supplementary file 2 — (DOCX 31.7 kb) [file 11764_2017_603_MOESM2_ESM.docx]

| No | Reference | Country | Population | Number of participants | Age  range | Diagnosis | Method of treatment | Research Method | CASP Quality score |
| --- | --- | --- | --- | --- | --- | --- | --- | --- | --- |
|  | Beitz JM, Zuzelo PR. The lived experience of having a neobladder...including commentary Artinian BM and Watson LA with author response. Western Journal of Nursing Research. 2003; 25(3):294-321 28p. | Pennsylvania | Men and women who have a neobladder | 14, 10 males, 4 females | 48-71yrs mean 60.85 | Muscle invasive Bladder cancer | Neobladder surgery | Narrative description of the lived experience of having a neobladder - interviews | 9/9 |
|  | Berry DL, Nayak M, Halpenny B, Harrington S, Loughlin KR, Chang P, et al. Treatment Decision Making in Patients with Bladder Cancer. Bladder Cancer. 2015;1(2):151-8. | USA | Patients diagnosed with bladder cancer of any stage and making a treatment decision in the last 6 months | 60, 45 men and 15 women | 33-86 | Bladder cancer , 28 with NMIBC, 18 MIBC, stage II-III, 14 with stage IV metastatic disease | Neoadjuvant Chemotherapy  Systemic chemotherapy  Neobladder  Transurethral resection  Cystectomy  Radiation therapy  Urinary diversion to a stoma (bag) | Semi structured interviews | 8/9 |
|  | Cerruto MA, D'Elia C, Cacciamani G, De Marchi D, Siracusano S, Iafrate M, et al. Behavioural profile and human adaptation of survivors after radical cystectomy and ileal conduit. Health and Quality of Life Outcomes. 2014; 12(1). | Italy | Patients with urinary diversions (radical cystectomy / ileal conduit | 30, 17 males, 13 females | Male mean age – 76.4yrs females 77.1yrs | Bladder cancer | Radical cystectomy with ileal conduit | Biographical approach guided by principles of grounded theory – narrative based interview | 5/9 |
|  | Clark M, Harris N, Martin S, Bartley K, DeBusk K, Abidoye O, et al. The impact of non-muscle invasive bladder cancer: qualitative research with patients. ISPOR 18th Annual European Congress; Milan, Italy2015. | USA | Patients with high risk non muscle invasive bladder cancer | 10, 5 men and 5 women | Mean age 73.1yrs (66-89) | NMI Bladder cancer | 3 treatment groups:  BCG naïve, treated with TURBT & chemotherapy, BCG relapse – due to be treated with cystectomy  BCG Refractory (failure to reach disease free state in 6/12 - also non improving or worsening of disease) or resistant to treatment | Semi structured interviews | 5/9 |
|  | Fitch MI, Miller D, Sharir S, McAndrew A. Radical cystectomy for bladder cancer: a qualitative study of patient experiences and implications for practice. Canadian oncology nursing journal = Revue canadienne de nursing oncologique. 2010; 20(4):177-87. | USA | Patients who have undergone radical cystectomy for bladder cancer 12 to 36 months previously | 22, 13 males and 9 females | 44-85 | Bladder cancer | Neobladder or ileal conduit, neobladder converted to ileal conduit (no continent reservoir) | Interviews and focus group | 9/9 |
|  | Foley KL, Farmer DF, Petronis VM, Smith RG, McGraw S, Smith K, et al. A qualitative exploration of the cancer experience among long-term survivors: Comparisons by cancer type, ethnicity, gender, and age. Psycho Oncology. 2006; 15(3):248-58. | USA | Long term survivors of bladder, breast, rectal, head and neck, cervix and prostate | 58 (6 = bladder). 32 males and 26 female overall, unknown as to sex of bladder cancer patients | 25-86 (all survivors) | Cancer - bladder, breast, rectal, head and neck, cervix and prostate | Unknown due to mixed cancer type | Semi structured interviews | 7.5 /9 |
|  | Hilton E, Henderson L. Lived female experience of chronic bladder cancer: a phenomenologic case study. Urologic Nursing. 2003. | USA | Female chronic bladder cancer patient undergone surgery to re establish urinary continence after cystectomy | 1 female | Doesn’t state | Bladder cancer | Surgery to re establish urinary continence after cystectomy | Hermeneutic phenomenology – interview with patient, researcher reflexivity and journal notes | 5/9 |
|  | Kowalkowski MA, Chandrashekar A, Amiel GE, Lerner SP, Wittmann D, Latini DM, et al. Examining Sexual Dysfunction in Non-Muscle-Invasive Bladder Cancer: Results of Cross-Sectional Mixed-Methods Research. Sexual Medicine. 2014; 2:141-51. | USA | Bladder cancer survivors | 26, 4 female, 22 males | Mean 69.1yrs | NMIBC, | Doesn’t explicitly state but analysis reports a mixture of cystectomy, BCG, | Survey and separate sample were interviewed (semi structured interviews) | 7/9 |
|  | Mohamed NE, Chaoprang HP, Hudson S, Revenson TA, Lee CT, Quale DZ, et al. Muscle invasive bladder cancer: Examining survivors’ burden and unmet needs. The Journal of urology. 2014; 191(1):48-53. | USA | Patients MIBC | 30, 22 male, 8 female | Mean age of 67 | Muscle Invasive bladder cancer | Cystectomy and urinary diversion | Semi structured Interviews (face to face or telephone) | 7/9 |
|  | Perlis N. Developing the Bladder Utility Symptom Scale: A Multiattribute Health State Classification System for Bladder Cancer: University of Toronto; 2013. | USA | 1)NMIBC with or without Intravesical therapy,  (2) MIBC treated with cystectomy and urinary reconstruction,  (3) MIBC treated with chemoradiation therapy, and  (4) Locally advanced or metastatic BC. | 31 patients in focus group  16 telephone interview  34 men, 13 women | Mean 66yrs | Only 4 patients had metastatic disease, all of which  were diagnosed during surveillance following radical intervention. Ten patients had NMIBC  while the remaining 36 had MIBC, 10 of whom were treated primarily with radiation therapy and  the remainder with surgical approaches. Twenty-two patients underwent radical cystectomy with  various urinary diversions: 13 ileal conduits, 6 neobladder, 2 ureterosigmoidostomies, and 1 continent reservoir. | Radical cystectomy  Intravesical therapy  Systemic chemotherapy | Focus groups (4, ) or telephone interviews to develop items in Bladder Utility Symptom Scale | 6/9 |
|  | Persson E, Hellstrom AL. Experiences of Swedish men and women 6 to 12 weeks after ostomy surgery. J Wound Ostomy Continence Nurs. 2002; 29(2):103-8. | Sweden | Patients who had stoma operation | 9, 2 males were bladder cancer patients who had experienced a urostomy | 44-67 | 2 Bladder cancer, 7 various other cancers | urostomy | interviews | 9/9 |
|  | Skea Z, MacLennan S, Entwistle V, N'Dow J. Communicating good care: A qualitative study of what people with urological cancer value in interactions with health care providers. European Journal of Oncology Nursing. 2014; 18(1):35-40. | UK | Patients with urological cancer | 26, 20 men, 6 women , of those 6 had bladder cancer (2 female, 4 male) | 37-80 | Bladder cancer and other cancer (prostate, kidney and testes | unknown | Telephone interview | 8/9 |
|  | Treanor C, Donnelly M. Late effects of cancer and cancer treatment—the perspective of the patient. Supportive Care in Cancer. 2016;24(1):337-46. | UK | Cancer survivors who reported moderate or severe late effects during a survey | 16, 3 of which have urinary cancer ( 1 female, 2 male) | 54-59 (urinary cancer only) | Urinary cancer | unknown | Semi structured interview | 8/9 |
|  | Williams-Cox D. A mixed-method study into quality of life for bladder cancer patients. Prof Nurse. 2004; 19(6):343-7. | UK | Bladder cancer patients who have undergone Intravesical therapy | 13, 12 male, one female | Intravesical TX – mean age 64yrs , maintenance 65.2yrs | Transitional cell carcinoma bladder cancer | Intravesical therapy – immunotherapy BCG, chemotherapy mitomycin, maintenance treatment | Mixed methods - Case studies – 2 patients (newly diagnosed and maintenance therapy), interviews, survey | 5/9 |
