# Supplementary material for: The patients’ experience of a bladder cancer diagnosis: a systematic review of the qualitative evidence
Source: J Cancer Surviv. 2017 Feb 17;11(4):453–61. doi: 10.1007/s11764-017-0603-6 (PMC5500680; doi:10.1007/s11764-017-0603-6)
Supplement: Supplementary file 3 — (DOCX 34.0 kb) [file 11764_2017_603_MOESM3_ESM.docx]

Online Resource 3: Preliminary Conceptual Framework of the patients’ experience of being diagnosed with bladder cancer through to survivorship

| Speed and momentum of diagnosis and treatment can result in patients feeling ill prepared, in particular for the side effects of treatment | | | | | | | | | | | | | | | | | |
| --- | --- | --- | --- | --- | --- | --- | --- | --- | --- | --- | --- | --- | --- | --- | --- | --- | --- |
|  | **Diagnosing Bladder Cancer** | | |  | **Patients experience of acute care and treatment** | | | |  | **Patients experience post (surgical) treatment** | |  | **The New Normal** | | | | |
|  | Physical Symptoms | Emotional Response | Cognitive Response |  | Preparing for surgery | | | Non-surgical treatment |  | **Becoming a cancer survivor :**  ‘an unfolding path’ | |  | Fluctuating Impact on QoL | | | | |
|  |  |  |  |  | Psychological  ‘Metamorphosis’ | | Physical | Physical  (temporary pain) |  |  |  |  | Negative | | | Positive | |
|  |  | | |  |  | | | |  | **Immense change:**  Adapting to new, distressing experiences | |  | Poor sleep. dependence, worry (leakage / odour), decline in social activities, not being the same, decreasing optimism, loss of friends, lack of sexual activity, hernia’s, skin lesions | | | Greater appreciation for life | |
|  | **Treatment Decision Making** | | |  | **Waking up after surgery**  ‘Alienation from the body’ | | |  |  | Psychological | Physical |  | **Permanent incontinence** | | | | |
|  |  | | |  |  | | |  |  | Acceptance of reduced pace of living | Long recuperation |  | New toileting characteristics (incl. bowels) | | Subsequent adaptions returning to work | | Reluctance to travel (avoid public toilets) |
|  |  |  |  |  |  |  |  |  |  |  | Weight loss |  |  |  |  |  |  |
|  | Active | Shared | Passive |  | **Post-operative care** | | |  |  | Unhappiness with lost vitality | Exhaustion and weakness |  | **Changing Sexuality**  **‘a shock’** | | | | |
|  |  |  |  |  |  |  |  |  |  |  |  |  | Surgical | | | Non-surgical | |
|  | Influences  (survival statistics, treatment preference, age, level of recreational and work activity) | |  |  | Pain management | Training  (stoma appliances and catheters) | |  |  | Lack of support (friends / family) | Variable homecare provision (access and expertise) |  | Permanent abstinence | | | Temporary abstinence (fear of contamination) | |
|  |  |  |  |  |  |  |  |  |  |  |  |  | Men  Impotence  Loss of manhood | Women  Vaginal dryness  Loss of intimacy  Appearance of stoma / fear of leakage was off-putting | |  |  |
|  |  | | |  |  | | | |  | **Management:** | |  | Age, stage of life, and how much importance a couple placed on sex determined acceptance | | | | |
|  |  |  |  |  |  |  |  |  |  | Self –initiated coping strategies | Physical routines to regain strength |  | **Living with the lifelong threat of cancer**  deal with it’ and ‘just take it as it comes’ | | | | |
|  | **What’s important to the patient** | | |  | **What’s important to the patient** | | | |  | **What’s important to the patient** | |  | **What’s important to the patient** | | | | |
|  | - Open communication - interpersonal aspects of care - treated as someone who matters - recognised and responded to as a unique individual - Understanding of impact of treatment options on the patient’s life. - Treatment plans to be clear, timely and consistent | | |  | - Simple acts of kindness – holding their hand as they wake from surgery - Training to continue after discharge - Effective pain management | | | |  | - Support with post-surgery recuperation (aftercare and expertise) - Support of family and friends | |  | - support from family and friends is vital throughout their (ongoing) journey with cancer - Good communication between partners helps re-establish a sexual relationship after BC | | | | |
